# Biological and bionic hands: natural neural coding and artificial perception

**DOI:** 10.1098/rstb.2014.0209

**Published:** 2015-09-19

**Authors:** Sliman J. Bensmaia

**Affiliations:** Department of Organismal Biology and Anatomy, University of Chicago, Chicago, IL 60637, USA

**Keywords:** neuroprosthetics, somatosensory cortex, touch, proprioception, biomimicry

## Abstract

The first decade and a half of the twenty-first century brought about two major innovations in neuroprosthetics: the development of anthropomorphic robotic limbs that replicate much of the function of a native human arm and the refinement of algorithms that decode intended movements from brain activity. However, skilled manipulation of objects requires somatosensory feedback, for which vision is a poor substitute. For upper-limb neuroprostheses to be clinically viable, they must therefore provide for the restoration of touch and proprioception. In this review, I discuss efforts to elicit meaningful tactile sensations through stimulation of neurons in somatosensory cortex. I focus on biomimetic approaches to sensory restoration, which leverage our current understanding about how information about grasped objects is encoded in the brain of intact individuals. I argue that not only can sensory neuroscience inform the development of sensory neuroprostheses, but also that the converse is true: stimulating the brain offers an exceptional opportunity to causally interrogate neural circuits and test hypotheses about natural neural coding.

## 1. Introduction

Imagine you could move your body—your arms, your legs, your torso—but anytime your skin came into contact with something, you would not feel it. If you closed your eyes for any length of time, you would lose track of where your arms and legs were. Anytime you grasped an object, you would not be sure it remained in your hand without looking. And you would miss out on the important affective experience that is to touch the ones you love and to be touched by them. This mental exercise highlights the importance of our senses of touch and proprioception in everyday life.

Tetraplegia is a devastating condition—typically caused by an upper spinal cord injury or by a disease of the nervous system—in which the body below the neck is no longer connected to the brain. As a result, tetraplegic patients can no longer move nor feel their bodies below the neck. One way to restore some independence for these patients is to equip them with anthropomorphic robotic arms [1], controlled by reading out signals from the motor areas of the brain. Indeed, the areas of the brain that sent signals to the arms before injury are spared and intended movements can be decoded from the neuronal activity in these motor areas ([2,3], see [4] for a review). However, the functionality of these neuroprostheses will be limited if they do not also provide for somatosensation. Indeed, in individuals with intact arms and motor pathways, but without somatosensory feedback, movements are slow, clumsy and effortful [5,6]. Furthermore, thought-controlled neuroprostheses without somatosensory feedback are experienced by patients as disembodied robots, despite the fact that they can control them by mere thought (Jennifer Collinger 2015, personal communication), which will limit their adoption. Finally, tetraplegic patients often yearn for the opportunity to touch loved ones, an experience that motor neuroprostheses currently do not afford.

**Figure 1.**
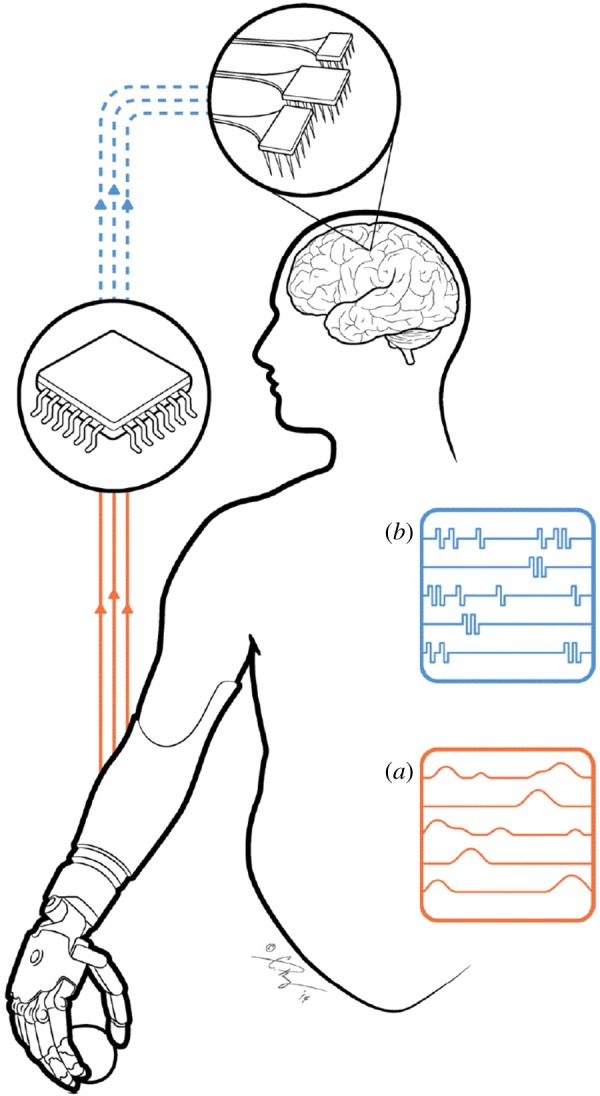
Diagram of a somatosensory neuroprosthesis. Signals from sensors on the prosthetic hand (*a*) are converted into trains of intracortical microstimulation (ICMS) delivered to S1 (*b*), designed to elicit meaningful sensations that convey information about objects grasped in the hand.

One way to restore somatosensation in tetraplegic patients is to electrically stimulate neurons in the somatosensory parts of the brain (figure 1). Indeed, the area of the brain that received sensory signals from the arm before injury—namely primary somatosensory cortex (S1)—is also spared. In brief, S1 comprises four different modules—Brodmann's areas 3a, 3b, 1 and 2—each of which exhibits somewhat different properties [7]. Neurons in area 3a respond primarily to movements of the joints, neurons in areas 3b and 1 respond to light touch, neurons in area 2 respond to both touch and proprioception. In principle, it should be possible to evoke meaningful sensory percepts by electrically activating somatosensory neurons. In attempting to do so, we can leverage decades of research investigating how information about touch is encoded in the brains of healthy individuals and attempt to reproduce patterns of brain activation that would be naturally evoked during everyday interactions with objects. To the extent that we are successful, these artificial somatosensory percepts will be verisimilar or at least informative. In our quest to restore touch using this biomimetic approach, we will be poised to test hypotheses about how the brain naturally encodes stimulus information. Thus, basic science will inform the development of next-generation neuroprostheses and neuroprosthetics will lead to new insights into basic science.

## 2. Two key precursors to somatosensory neuroprostheses

**Figure 2.**
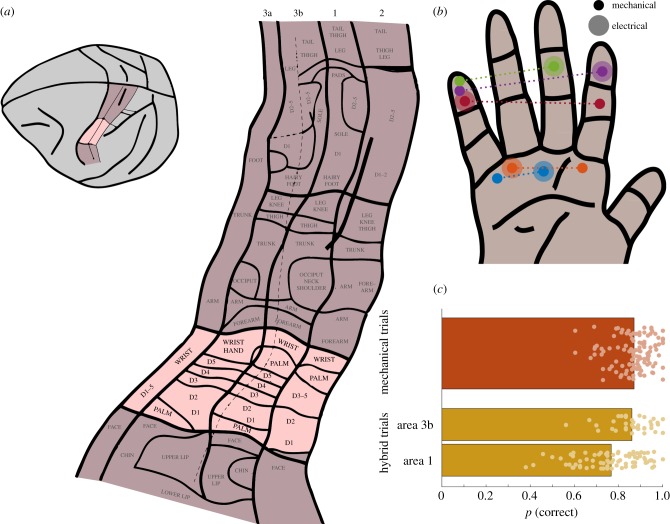
(*a*) Somatotopic organization of primary somatosensory cortex with the hand region highlighted (adapted from [9]). (*b*) Combination of skin locations at which pokes were delivered as well as receptive fields of electrodes through which stimulation was delivered. The dotted lines link conditions that were paired in a trial. As can be seen, each electrode replaced a poke; that is, the receptive field of each stimulated electrode corresponded to one of the poke locations. (*c*) Performance on mechanical and hybrid trials. Each dot represents a condition, bars represent the mean performance (adapted from [10]).

Beginning in the 1930s, Wilder Penfield and colleagues electrically stimulated the surface of the brain in search of the focus of pharmacologically intractable epilepsy—the area of the brain wherefrom it originates. The idea was that electrical stimulation of the epileptic focus would induce a seizure. When Penfield stimulated the surface of S1 [8], subjects reported tactile sensations that were localized to specific locations on the body. These electrically induced sensations were typically described as numbness, tingling and sometimes pain. Importantly, the projected location of these sensations varied systematically with the location of the stimulating electrode, leading to the discovery that somatosensory cortex is somatotopically organized (see figure 2*a* for the macaque homunculus).

In Penfield's studies, and others in which the surface of the S1 was stimulated (e.g. [11]), the evoked percepts were relatively diffuse and consisted primarily of paresthesias, which did not bode well for using electrical stimulation of cortex as a means to evoke meaningful percepts. The outlook improved considerably in the wake of a series of landmark experiments in the laboratory of Ranulfo Romo [12,13]. The team had taught animals to discriminate vibratory stimuli delivered to their fingertips. The animals' task was to judge which of two vibrations was higher in frequency. Romo showed (as Mouncastle had before him, see [14]) that skin vibrations evoke entrained responses in S1; that is, each stimulus cycle evokes a burst of spikes, which in turn results in a highly periodic neuronal response [15]. Romo speculated that it was this patterning in S1 responses that carried the information about vibration frequency. He tested this hypothesis by having the animals perform the frequency discrimination task based on intracortical microstimulation (ICMS) rather than skin vibrations: having learned to perform the vibratory discrimination task described above, animals were presented with two electrical pulse trains of different frequency, and their task was to judge which of the two trains was higher in frequency [12]. As each pulse evokes a burst of activity in cortex [16], pulse trains will evoke entrained responses whose frequency matches that of the pulse train. If indeed entrainment conveys information about frequency, then discriminating the frequency of ICMS pulse trains should be analogous to discriminating the frequency of skin vibrations, since both evoked periodic responses in S1 neurons. Romo showed that the animals' performance on the task based on ICMS was comparable to that based on skin vibrations. In fact, the animals could compare the frequency of an ICMS train to that of a skin vibration [13]. These results were significant for two reasons. First, while previous studies had revealed a correlation between temporal patterning in S1 and vibratory frequency (e.g. [15]), the study showed that this patterning conveys information about frequency using a causal manipulation of neuronal activation. Second, Romo and colleagues demonstrated that animals were able to perform a perceptual discrimination task based on artificially generated sensations. The trick had simply been to replace the natural afferent input with ICMS that mimicked the relevant aspect of neuronal activation, in this case neuronal entrainment. The landmark study demonstrated that artificial somatosensory percepts with specific properties could be elicited using ICMS, setting the stage for the development of somatosensory neuroprostheses.

## 3. The birth of somatosensory neuroprostheses

Two major innovations revolutionized neuroprosthetics in the twenty-first century [4]. The first is the development of anthropomorphic robotic limbs that reproduce much of the function of a native human arm [1]. The second is the development and refinement of algorithms that decode intended movements from the neuronal activity measured in motor areas of the brain. Together, these innovations make it possible for a human patient to move a robotic arm by thought alone [2,3]. Because these bionic arms can move in most of the ways that a biological one can, to control the bionic arms requires somatosensation for the same reasons that controlling a native one does: visual feedback is a poor substitute for its somatosensory counterpart when it comes to using hands to manipulate objects. Furthermore, somatosensation is required for embodiment and allows for affective communication. The importance of touch in everyday life spurred efforts to develop ways to restore it artificially.

The approach to restoring touch, then, consisted in delivering trains of electrical pulses to the somatosensory areas of the brain in the hopes of eliciting percepts that convey information about grasped objects. Early examples of somatosensory neuroprostheses demonstrated that animals could perform behavioural tasks based on largely arbitrary patterns of ICMS delivered to S1 [17–20]. These studies applied Romo's finding—that animals could discriminate ICMS that varied in frequency—to establish a proof-of-principle that this approach might be applied to bi-directional brain–machine interfaces—i.e. neuroprostheses that are under brain control and provide sensory feedback [20]. In fact, not only could animals discriminate ICMS pulse trains that varied in frequency, but they could also distinguish ICMS that was delivered to different neuronal populations [21]. An important commonality between the studies mentioned above is that the ICMS was not designed to evoke naturalistic patterns of neuronal activation. Rather, the idea was to create a systematic mapping between sensory events and ICMS such that the animal learned to use them to guide its behaviour.

Another approach to eliciting artificial percepts, and the one that we will be exploring in depth here, consists in leveraging what is known about how the brain encodes somatosensory information, and attempting to reproduce the relevant pattern of activation in the hopes of conveying this information intuitively and perhaps even of eliciting verisimilar sensations. On the face of it, this approach seems doomed to failure. First, while sensory neuroscience has made huge strides in the past 50 years, there is still much about neural coding that we do not understand. We cannot hope to reproduce relevant patterns of neuronal activation if we are not sure what those patterns are. Second, ICMS is a blunt tool: it produces unnaturally synchronized patterns of activation that one would never observe in a healthy brain [16]. Furthermore, it excites neurons of various types, most near the electrode but some far away [16,22,23], so the neural consequences of ICMS applied to a given volume of tissue are largely unknown. In the light of the seeming hopelessness of biomimetic ICMS, Romo's study is that much more significant. And so, inspired by Romo's success, we pursued the biomimetic approach to artificial somatosensation.

## 4. Exploiting the somatosensory homunculus

To grasp an object, it is critical to know which parts of the hand make contact with the object. Minimally, the thumb and one of the fingers have to make contact before it can be picked up. How is contact location encoded in the brain? As mentioned above, Penfield discovered that stimulating the surface of primary somatosensory cortex elicits sensations on the body surface that are somewhat localized. Furthermore, systematically changing the location of the electrode on S1 resulted in somatosensory percepts that shifted systematically across the body surface. As the electrode proceeded away from the midline, sensations progressed from the legs, to the trunk, to the arm, to the hand, to the face. Single-unit recordings in S1 have since revealed a systematic, somatotopic organization: the population of neurons that respond to the little finger is always medial and posterior to the population of neurons that respond to the ring finger, which in turn is medial and posterior to the population of neurons that respond to the middle finger, etc. (figure 2*a*). This organization is consistent across four complete body maps in S1, one in each of its modules (areas 3a, 3b, 1 and 2). Thus, when the index fingertip comes into contact with an object, a specific population of neurons becomes activated. When the thumb makes contact with an object, a nearby, largely non-overlapping population of neurons becomes activated. One might hypothesize, then, that contact location is encoded spatially in S1: the location of the activated neuronal population will determine the location of the evoked percept. Indeed, that is what Penfield found when stimulating the surface of the brain. The question, though, is whether artificial percepts could be sufficiently and repeatably localized for this principle to be used to convey information about contact location in a neuroprosthesis. Indeed, as discussed above, surface stimulation of S1 causes very diffuse sensations, probably owing to the fact that it excites diffuse populations of neurons. The development of invasive neural interfaces opened the possibility, then, that smaller populations of neurons could be stimulated, leading to more localized percepts.

One of the challenges facing the biomimetic approach to artificial somatosensation is that it requires that specific properties of artificial sensations be probed. With humans, this would be trivial: one would simply stimulate and have the subject report the resulting sensation, as Penfield did. To date, however, no human patient has ever been implanted with an invasive neural interface in S1. Even when it does happen (and it is only a matter of time at this point), time with the patient is at a premium, and it is virtually impossible to develop a new technique based solely on human testing. It is therefore necessary to develop approaches based on animal studies. To probe the quality of sensations in animals is challenging and requires that we train them to perform tasks that will allow us to infer what they feel.

To test the localization of ICMS-elicited sensations, we trained animals to discriminate the location of pokes applied to the skin [10,24]. The animals were sitting in front of a computer monitor that conveyed information about the flow of each experimental trial with their hand fixed palmar surface facing up. On each trial, two pokes were sequentially delivered to two locations on the palmar surface of the skin using a high-precision robot (figure 2*b*). The animal's task was to report whether the second poke was to the right or the left of the first. After a few months, the animals were able to perform this task accurately. The animals were then implanted with electrode arrays in the hand representation of S1 (areas 3b and 1; pink-shaded region in figure 2). For each electrode, we mapped receptive fields by delivering pokes across the surface of the hand and measuring the evoked neural activity. We could then determine to which patch of skin each electrode was most sensitive. Using these maps, we had the animal perform a modified version of the location discrimination task described above where, on some trials, we replaced one of the two pokes with ICMS to an electrode with the corresponding receptive field (figure 2*b*). For example, on one trial (a so-called mechanical trial), we might poke the index fingertip then the middle fingertip of the left hand, leading to a ‘left’ response. On the following trial (a so-called hybrid trial), we might poke the middle fingertip then electrically stimulate through an index fingertip electrode; we hoped that the second stimulus would be experienced as a virtual poke on the index fingertip. On that trial, then, the correct answer was ‘right’. Importantly, mechanical trials were interleaved with hybrid trials, and a variety of skin locations and electrodes were used in each experimental block. Thus, the animal could not learn to associate sensations with responses. It could only do the task based on the perceived location of pokes and virtual pokes.

We found that the animal performed both the mechanical and hybrid tasks well above chance (figure 2*c*). In fact, its performance on hybrid trials was nearly as good as that on mechanical trials when the ICMS was delivered to area 3b. Furthermore, the animal's performance was almost at asymptotic levels on the very first day that hybrid trials were introduced, further demonstrating that it was performing the task based on the location of virtual pokes rather than by learning arbitrary stimulus–response pairings. These experiments led to the conclusion that ICMS of S1 through individual penetrating electrodes results in highly localized tactile percepts. From a scientific standpoint, this result confirms the hypothesis that contact location is encoded spatially in S1; that is, perceived location is determined by *where* across the surface of S1 the evoked activity is located. From a neural engineering standpoint, the results provide a blueprint on how to convey information biomimetically about contact location in a neuroprosthesis.

Imagine that a tetraplegic patient has been implanted with an array of electrodes in the hand representation of S1, which can be found based on anatomical markers [25]. We now know that if we stimulate through an electrode on this array, the patient will experience a tactile sensation on a specific region of his or her hand. Now, suppose that stimulation through electrode 53 elicits a percept on the thumb tip. We can then connect the sensor on the prosthetic thumb to electrode 53. Anytime the thumb touches something, ICMS will be delivered through electrode 53, and a sensation will be elicited on the thumb, thereby signalling the contact location in an intuitive way. We are exploiting the organization of the brain to intuitively convey information about a specific stimulus property, namely its location on the skin.

## 5. Embodiment

One might argue that, when the prosthetic thumb touches an object (thus resulting in stimulation through electrode 53), the evoked sensation will be on the tetraplegic patient's native thumb, not the prosthetic one. The patient still has to make an association between locations on his or her skin and locations on the prosthetic hand. That the locations are matching might make this mapping somewhat more intuitive, but is it worth all of this trouble? Evidence suggests that, with time, the sensations might migrate to the prosthetic hand. In other words, the patient may embody the prosthetic limb and start to feel as though the robot is part of his or her body! To understand how this might happen, we need to examine a phenomenon called ‘the rubber hand illusion’ [26]. In an example experiment, a fake arm is placed in front of a subject in a natural posture, while the native arm is hidden from sight. Then, the experimenters pair visually experienced pokes delivered to the fake hand with matched, felt pokes delivered to the real hand. After a few repetitions, subjects begin to experience the rubber hand as their own (even though they know that it is not). The sense of embodiment is so vivid that, if someone injures the fake hand, subjects exhibit a fear reflex as if their native hand had been injured [27]. Thus, when visual experience of contact is paired with spatially congruous tactile experience of contact, patients begin to embody the extra-corporeal limb.

The rubber hand illusion has been exploited to help amputees embody prosthetic limbs, as illustrated in an experiment with a population of patients who had undergone a procedure called targeted muscle reinnervation (TMR) [28]. In this technique, the residual nerves that used to innervate the now missing distal limb are rerouted to the pectoral muscle. After the surgery, nerve fibres begin to invade and make connections with the muscle and the overlaying skin. Motor commands that were intended to move an amputated digit, say, resulted in contractions of patches of pectoral muscle. Signals from sensors placed on this muscle can then be used to move that digit in the prosthetic hand. At the same time, touching patches of skin on the chest evokes sensations that are projected to the amputated arm. Indeed, these skin patches are innervated by fibres that used to innervate the arm so the resurrected signals originating from these fibres are interpreted as stemming from the missing limb. The experiment, then, consisted in touching an unseen patch of chest skin while poking a (seen) fake limb at the location wherefrom the sensations seemed to originate [29]. After repeated tactile stimulation paired with congruous visual stimulation, the patient began to feel as though these tactile sensations originated from the limb (which, again, they knew to be fake). That is, they began to embody the arm. A similar effect was achieved when touches to the stump of amputees (who had not undergone TMR) were paired with seen pokes to a prosthesis [30]. While this phenomenon has not been investigated with artificial somatosensation delivered through a brain interface, the biomimetic mapping described above will likely lead to embodiment of the prosthetic limb (cf. [31]). Indeed, patients will experience concurrent and congruous visual sensations, the necessary preconditions to achieve embodiment. The neuronal tissue that once represented the biological limb will be fully appropriated by the bionic one.

## 6. Feeling pressure

**Figure 3.**
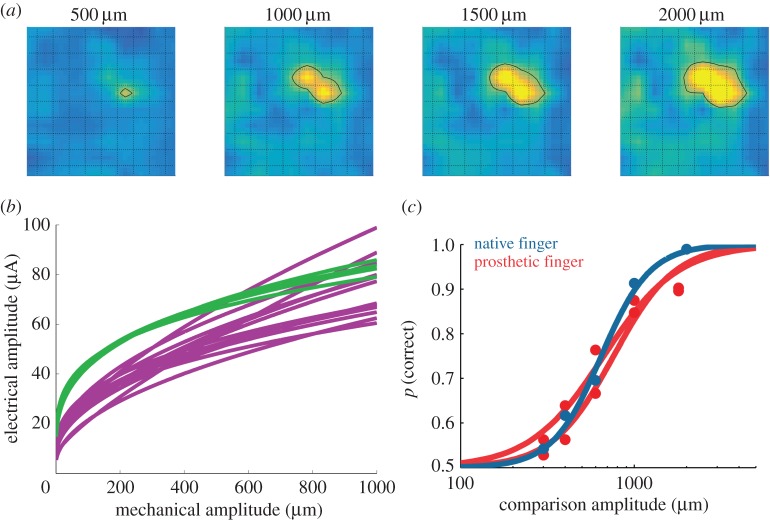
(*a*) Neuronal activation evoked over a 4 **×** 4 mm patch of cortex by indentations delivered to the tip of the little finger at four amplitudes. As the amplitude increases, the firing rate increases and the area of activated neurons also increases. (*b*) Psychometric equivalence functions that map electrical amplitude onto mechanical amplitude such that the ICMS and the corresponding poke are of equal perceptual magnitude (each curve corresponds to one electrode/skin location pair; different colours denote different animals; reproduced from [10]). (*c*) Animals perform identically on a pressure discrimination task whether pokes are delivered to their native finger (blue) or to a prosthetic one (red). The standard amplitude for these experiments was 150 µm (reproduced from [10]).

When we grasp an object, we minimally need to know not only which fingers are in contact with it, but also how much pressure we are exerting on it. We need to apply just enough pressure so that the object does not slip from our grasp when we pick it up but not so much that we crush it. Tactile signals convey very precise information about contact pressure [32]. In primary somatosensory cortex, an increase in pressure results not only in an increase in the activity of the neurons that are most sensitive to the skin location at which it is applied (neurons in the hotzone of activation) but also in the recruitment of nearby neurons (figure 3*a*). A biomimetic approach to conveying information about contact pressure would then be to modulate both the firing rate of the neurons in the hotzone and the volume of neurons activated in responses to changes in skin pressure. It turns out that we can achieve both of these changes in neuronal activity by modulating the ICMS amplitude [16,23]. Indeed, increases in current amplitude will result in stronger depolarization of nearby neurons, thereby increasing the strength of their response, and in greater current spread, leading to the recruitment of more distant neurons.

To test the hypothesis that changes in ICMS amplitude could be used to convey information about contact pressure, we trained animals to perform both a tactile detection task and a pressure discrimination task [10,24]. On each trial of the detection task, a poke was presented in one of two stimulus intervals and the animal reported in which of the two intervals it occurred (by saccading to one of two visually presented targets). As might be expected, the animal's performance improved as the amplitude of the poke increased. On each trial of the discrimination task, two pokes were delivered to the same location on the hand and the animal reported which of the two was stronger. As the difference in amplitude between the two pokes increased, the animal's performance improved. Once animals were trained on the task, we had them perform the same tasks, except that the pokes were replaced by ICMS. As had been the case with the location discrimination task described above, the animals were immediately able to perform the detection and discrimination tasks based on ICMS after having been trained with pokes. This immediate transfer provided an initial clue that the sensory correlates of changes in contact pressure and in electrical amplitude were at least somewhat comparable. On each day, the animals would perform the detection and/or discrimination task with pokes on the hand followed by the same task with electrical stimuli applied to an electrode whose receptive field matched the poked skin location.

From these data—mechanical and electrical amplitude detection and discrimination performance—we derived a sensory encoding function that mapped the pressure exerted on the skin onto a perceptually equivalent ICMS pulse train. This mapping was achieved by assuming that stimuli that were equally detectable were perceptually equivalent. In other words, if a 150 μm poke on the index fingertip and 30 μA ICMS to an electrode with a receptive field on the index fingertip were both detected 75% of the time, we assumed that their sensory magnitude was the same. Then, if a 500 μm poke was discriminable from the 150 μm poke 75% of the time and the 60 μA was discriminable from the 30 μA ICMS 75% of the time, then 500 μm and 60 μA were equivalent, and so on. The resulting function converted pressure to ICMS amplitude such that the perceptual magnitudes of the evoked sensations were equal in the two modalities, precisely the kind of mapping we need in a prosthesis (figure 3*b*).

We then tested this sensory encoding function in two ways. First, we had animals perform a hybrid pressure discrimination task. In this task, a mechanical stimulus that was matched in perceived magnitude with 50 μA ICMS was paired with ICMS that ranged from 20 to 80 μA. As predicted from our sensory encoding function, if the ICMS was less than 50 μA, the animal judged it as being weaker than the poke and vice versa, thereby validating the mapping. That the animals could do this at all was very promising because, once again, it suggested that skin pokes and ICMS could readily be compared. This is not to say that the artificial tactile sensations felt exactly like pokes, however, but real and virtual pokes fell on a common perceptual continuum. Second, we had the animals perform the mechanical detection and pressure discrimination task described above, except they felt the pokes through a prosthetic hand. Specifically, we used our indenting robot to poke the prosthesis with the same stimuli we had used in the tactile detection and pressure discrimination experiments described above. We then used our sensory encoding function to compute, in real time, the amplitude of ICMS from the time-varying output of the force sensors on the prosthetic finger. With our set-up, we were able to provide the animals, in real time, with biomimetic information about contact pressure through ICMS delivered to S1. In other words, we had implemented our sensory encoding function in a real neuroprosthesis. We found that the animals performed identically on detection and discrimination tasks whether the pokes were delivered to their native finger or to the prosthetic finger (figure 3*c*). In other words, our sensory encoding function performed exactly as we had hoped. Note that we could have easily made the animals better at pressure discrimination by making the function steeper, or worse by making it shallower. The consequence would have been to reduce or extend the range of discriminable pressure increments. The goal, however, was to evoke a sensory percept that was appropriate to the applied pressure; our sensory encoding function allowed us to do that by mimicking, at least qualitatively, the representation of pressure when natural afferent input is present. Again, that animals could so readily go back and forth between natural and artificial input supported the underlying neural coding hypothesis, which was that pressure is encoded in the increase in the activation of neurons at the hotzone and in the recruitment of nearby neurons that accompany an increase in applied pressure (figure 3). At the same time, the sensory encoding function provided a blueprint on how biomimetic information about pressure could be conveyed in a neuroprosthesis.

Of course, in a tetraplegic patient, sensory encoding functions cannot be developed for each matched electrode and skin location, as was done with the monkeys, whose nervous systems were intact. However, sensory encoding functions that were independently derived for different skin location/electrode pairs and from different monkeys were very similar. In a tetraplegic patient, then, we could use a canonical sensory encoding function—for example, the mean of all the functions we obtained from our monkeys—for all electrode/sensor pairings. We could calibrate this canonical function—make it steeper or shallower—by adjusting the ICMS level such that it perceptually matches a benchmark tactile stimulus (e.g. delivered to the face, where sensation is intact).

## 7. Timing contact

When we reach for an object, we preshape our hand to match the shape of the object. The instant we make contact with the object, we terminate our reach and complete our grasp. Precise information about when we make contact is important to avoid overreaching. The sensory encoding algorithm described above would not be well suited to signal contact events, because initial contact is characterized by very low pressure levels that increase over tens or hundreds of milliseconds [33]. Thus, by the time contact is signalled by the sensory encoding algorithm described above, it is too late and the hand has reached too far. In S1, both the onset and offset of contact are signalled by very precisely timed phasic responses, so-called on- and off-responses [32,34]. These responses originate from peripheral mechanoreceptors that are very sensitive to changes in applied pressure [35,36]. One way to restore precise timing information, then, is to deliver phasic ICMS pulses, lasting approximately 50–100 ms, at the onset and offset of contact to mimic the on- and off-responses that are ubiquitous in S1 [10]. The phasic ICMS at contact would then be followed by tonic ICMS that tracks the pressure applied to the object based on the sensory encoding algorithm. When the object is released from grasp, another phasic ICMS would be delivered to mimic the native off-response. Some evidence suggests that ICMS-evoked percepts are slower to guide behaviour than are their tactile counterparts [37], which may corrupt information about contact timing. Perhaps this sluggishness of artificial tactile percepts can be overcome by increasing the stimulation amplitude or delivering ICMS trains that more closely mimic natural patterns of neuronal activation. Either way, the functional benefits of these transient signals have not yet been tested. When we can compare the performance of an animal or a patient on a task that requires grasping and manipulating objects with a prosthetic hand with and without these biomimetic signals about contact timing, we will be in a position to determine not only the benefit of these signals to neuroprosthetics, but also the extent to which contact transients signal contact timing in intact individuals. Ultimately, such contact-related transients can be replaced with more sophisticated sensory encoding functions that not only take into consideration the instantaneous pressure that is applied to the skin (as does the sensory encoding algorithm described above), but also the rate of change in pressure. Such mappings would not only mimic natural on- and off-responses, but also accommodate the dynamics of responses to time-varying pressure that naturally occur during everyday interactions with objects.

## 8. Expanding the sensory repertoire

The experiments described above provide a blueprint on how to convey basic tactile information—about contact location, contact pressure and contact timing—through ICMS of S1. However, our sense of touch conveys much more elaborate information about objects: information about their size and shape, about their material properties and surface microstructure (texture), and about how they move across the skin [38]. In somatosensory cortex, different populations of neurons encode these different properties: some S1 neurons respond to the shape of objects by encoding the orientation of their edges [39]; others are sensitive to the curvature of object contours [40]; others are tuned for the direction in which the objects move across the skin [41]; others seem to encode coarse [42] and fine [43] surface microstructure. Higher-level object features seem to be encoded more explicitly at higher levels of cortical processing, with relatively non-selective responses in area 3b and stronger selectivity in areas 1 and 2. One possibility, then, would be to exploit feature-specific representations in somatosensory cortex to convey richer tactile information about objects.

In a landmark study investigating the neural basis of visual motion perception, Newsome and colleagues provided a first glimpse that this approach might work [44]. In this experiment, animals were presented with a visual display consisting of moving dots. A proportion of the dots tended to move in the same direction, while the rest moved in random directions. The animals' task was to judge in which of two directions the ensemble of dots was moving. The task was easy when all of the dots were moving in the same direction, but became more and more difficult as a greater proportion of the dots moved randomly. A subpopulation of direction-selective neurons had been identified in the medial temporal (MT) cortex, a visual area dedicated to motion processing. Newsome and colleagues then probed whether the perception of the dot patterns could be biased by stimulating direction-selective neurons. In other words, would the animals' tendency to perceive a display as moving leftward, say, increase if a neuron selective for leftward motion was stimulated? Indeed, they found that motion perception could be systematically biased by stimulating direction-selective neurons. The scientific implication of this study was that these direction-selective neurons in MT were causally involved in motion perception, extending beyond the standard correlational study linking neuronal response to stimulus properties. The implication for neuroprosthetics was that feature selectivity could be exploited to produce artificial sensations with specific qualities.

Indeed, we could convey information about the shape of objects by stimulating shape-selective neurons in somatosensory cortex. Simple edge detection algorithms [45] could be used to identify the orientation and curvature of object contours impinging on each fingertip. Then, S1 neurons selective for that particular orientation and curvature could be stimulated. In principle, at least, stimulation of these feature-selective neurons might evoke a percept of that feature. London and Miller have provided preliminary evidence that this approach might work in the context of a proprioceptive neuroprosthesis. Indeed, they identified a subpopulation of neurons in area 2 that are selective for the direction of limb motion in a manner that is qualitatively analogous to the visual motion-selectivity observed in MT [46]. Then, Miller and colleagues had animals perform the proprioceptive analogue of the visual task described above: the limb of the monkey was lightly bumped in one of two directions and the animal's task was to report the bump direction [47]. As was found in the visual experiments, when the physical bump was accompanied by ICMS applied through an electrode with known direction preference, the animals exhibited an increased tendency to judge the bump as having been in that direction. Critically, when ICMS was delivered without a physical perturbation, the animal tended to respond as if a bump had been delivered in the preferred direction of the stimulated neuron. On those trials, interleaved with the paired mechanical/electrical trials, the physical bump was entirely replaced with a virtual one, and the animal still responded as if a physical bump were presented. To the extent that this approach is robust, it could be used to convey information about all stimulus dimensions that are explicitly encoded in S1, including stimulus orientation/curvature, motion and texture. In a tetraplegic patient, the feature selectivity of neurons would not be characterized based on their responses to natural afferent input but rather based on the patient's reports of the evoked percepts when ICMS is delivered through the corresponding electrode.

Conveying information about basic sensory information (contact location, pressure, timing) and higher-level features might be achieved by stimulating different neuronal populations. Indeed, as mentioned above, feature selectivity is stronger at higher stages of cortical processing. Thus, one might convey basic contact information by stimulating low-level areas, say area 3b, and convey feature-specific information by stimulating higher areas, including areas 1 and 2 and even secondary somatosensory cortex. This approach might actually benefit from the fact that ICMS-induced neuronal activation does not seem to propagate normally through hierarchical networks of neurons. Indeed, activation of primary visual cortex by ICMS does not evoke excitatory activation in secondary visual cortex in the same way that natural afferent input does [48]. It may then be that ICMS of area 3b will only minimally interfere with ICMS of areas 1 and beyond. The power of this approach is that it exploits the processing that takes place in upstream circuits and culminates in explicit representations of behaviourally relevant stimulus features. Of course, this feature-based tiling of artificial touch faces significant technological challenges, some of which are discussed briefly below.

## 9. The problem of cortical plasticity

When afferent input from a limb is eliminated, the part of the brain that used to respond to that limb starts to respond to other body parts [49]. Typically, the invading signals stem from body regions with adjacent S1 representations (the face, the trunk) or that are overused to compensate for the missing limb [50,51]. If the brain is so malleable, one might expect that spinal cord injury or amputation would completely change the underlying neuronal representations. Does it even make sense to invoke a biomimetic approach to artificial touch if the brain is changing so much post-injury? It turns out that the reorganization of S1 after deafferentation is not as dramatic as it might seem. First, invading signals from other body regions reflect the unmasking of lateral connections at the level of the cuneate nucleus [52], rather than major structural and functional changes in deafferented cortex. Second, phantom hand ‘movements' evoke activity in the deafferented cortex [53,54]. Third, electrical stimulation of deafferented limb regions of S1 in human amputees evokes sensations on the phantom limb rather than on the invading body regions [55,56]. Thus, while deafferented cortex can be excited by other body parts, downstream cortical regions still interpret this activation as originating from the missing or deafferented limb.

The question remains whether the functional properties of these neurons, whose somatotopic organization seems to be rather stable, change after deafferentation. For example, does tuning for tactile motion direction disappear a year post-injury? If so, feature-specific representations cannot be exploited to expand the repertoire of tactile sensations, as sketched out in the previous section. While refined through sensory experience, feature selectivity is driven in part by endogenous mechanisms of development [57], so it likely reflects structural properties of cortex that are not so easily undone. While we will not know for sure until a deafferented human patient is stimulated intracortically in S1, it is likely that neuronal representations are stable enough to be exploited to achieve artificial touch.

## 10. Practical considerations

While the cutaneous representation of the hand is approximately 7 × 4 mm in monkeys, so can be covered using a small number of arrays, it is about 30 × 20 mm in humans. To blanket it would thus require tens of electrode arrays. Given the current state of the art in neural interface technologies, one might elect to target the fingertip representations, for example, by splitting one array five ways, with a relatively small set of electrodes impinging upon each digit representation. In fact, interfacing with just the thumb and index finger tips might go a long way towards improving the functionality of prosthetic hands. Such a modest number of electrodes will likely limit the spatial resolution of the artificial touch, and eliminate any possibility of restoring a wide range of tactile sensations. Regarding spatial resolution, a hard ceiling is set by the spread of stimulating current, which radiates out hundreds of micrometres to multiple millimetres away from the electrode tip over the detectable and safe range of amplitudes. In the experiments described above, we never tested whether two adjacent electrodes evoked spatially distinct percepts. Therefore, it is unclear whether the bottleneck on spatial resolution is current spread or electrode spacing.

The electrode density and coverage demands are considerably greater if one wishes to elicit percepts with different qualities at each location based on the feature selectivity of neurons, as discussed above. Indeed, this would require the ability to selectively activate neurons with different feature preferences (hopefully resulting in percepts with different qualities) but overlapping receptive fields.

In the light of these considerations, efforts are underway to increase the density of electrodes and their coverage [58,59]. Regardless, full sensory restoration is not necessary to achieve substantial function, as evidenced by the success and widespread use of the cochlear implant.

## 11. The future of somatosensory neuroprostheses

Brain interface technologies are still rather primitive, consisting of metal electrodes, which cause major inflammation and often fail catastrophically [59–64]. Electrical stimulation is a blunt tool that unselectively and synchronously activates neurons near the electrode tip. Nonetheless, these primitive tools have already been fruitfully used to evoke informative sensations. Electrodes will improve by becoming more biocompatible and compliant [59,65–69], thus less damaging to the neuronal tissue. Optogenetics will replace electrical stimulation, and thus allow for much more controlled and selective stimulation. As these technologies progress, artificial touch will increasingly mimic its natural counterpart. The hope is that a tetraplegic patient will one day be able to dexterously manipulate objects with an embodied robotic hand and regain that critical sensory experience that is touch.
